# Supplementary material for: MEGA12: Molecular Evolutionary Genetic Analysis Version 12 for Adaptive and Green Computing
Source: Mol Biol Evol. 2024 Dec 21;41(12):msae263. doi: 10.1093/molbev/msae263 (PMC11683415; doi:10.1093/molbev/msae263)

Supplementary Information

MEGA12: Molecular Evolutionary Genetic Analysis version 12 for adaptive and green computing

Sudhir Kumar[1,2,*], Glen Stecher[1], Michael Suleski[1], Maxwell Sanderford[1], Sudip Sharma[1,2], and Koichiro Tamura[3,4]

[1] Institute for Genomics and Evolutionary Medicine, Temple University, Philadelphia, PA 19122, USA

[2] Department of Biology, Temple University, Philadelphia, PA 19122, USA

[3] Department of Biological Sciences, Tokyo Metropolitan University, Tokyo, Japan

[4] Research Center for Genomics and Bioinformatics, Tokyo Metropolitan University, Tokyo, Japan

*Corresponding author: s.kumar@temple.edu

Supplementary Figure S1. Usage of *MEGA* for various analyses from January 2023 to September 2024. (***a***) Types of analyses. (***b***) Methods of phylogenetic inference. (***c***) Sequence alignment methods. (***d***) Testing of phylogeny. Trends shown are based on data collected using an in-built system to gather anonymous usage data from users who allow this data collection. If a user opts to share their usage data, some versions of *MEGA* save a report of the choices made in the *Analysis Preferences* dialog box. No information about the datasets analyzed is collected, nor is personal or computer information identified. This system is only contained in the GUI version of *MEGA* for the MS *Windows* operating systems, and only a tiny fraction of users permitted data collection. So, these counts are likely to be substantial underestimates of the actual counts of analyses conducted.

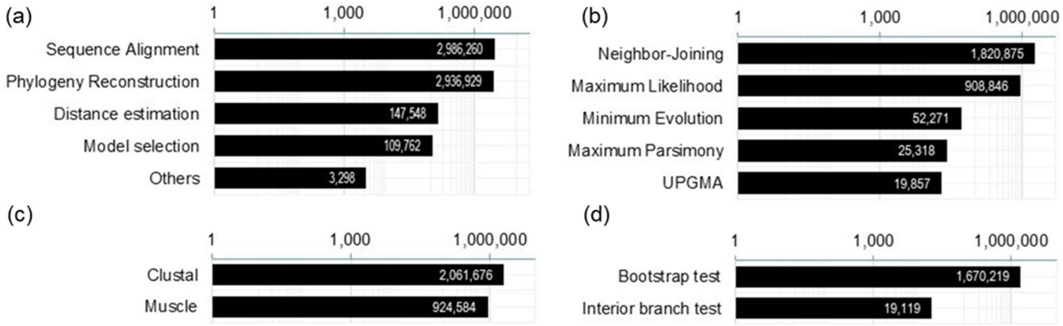

Supplementary Figure S2. Based on the information received when downloading, downloads, and users of MEGAX and MEGA11 from January 2023 to September 2024. Downloads of (***a***) GUI versions and (***b***) Command-line [CC] versions. (***c***) Types of institutions. (***d***) types of users. Data from Debian, RedHat, and other distributions of Linux are pooled together.

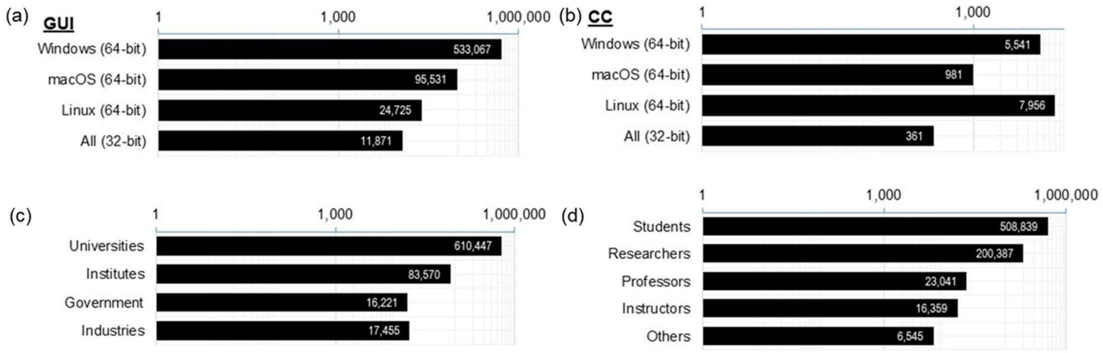

Supplement: msae263_Supplementary_Data [file msae263_supplementary_data.pdf]
